# Targeted therapy plus chemotherapy for advanced or metastatic triple-negative breast cancer: a PRISMA 2020 systematic review and meta-analysis

**DOI:** 10.3389/fonc.2026.1903922

**Published:** 2026-08-14

**Authors:** Yanbing Li, Zijun Li, Xin Shi, Hongbo Zuo

**Affiliations:** Department II of Oncology, Jiujiang City Key Laboratory of Cell Therapy, JiuJiang NO.1 People's Hospital, Jiujiang, Jiangxi, China

**Keywords:** advanced breast cancer, chemotherapy, objective response rate, safety, targeted therapy, triple-negative breast cancer

## Abstract

**Objective:**

To systematically review the efficacy of chemotherapy combined with targeted therapy in the handling of advanced triple-negative breast cancer (TNBC).

**Methods:**

A thorough literature search was carried out across several databases, including PubMed, EMBASE, ScienceDirect, and Chinese medical databases, for case-control trials on TNBC patients treated with chemotherapy combined with targeted therapy versus chemotherapy alone. Two separate researchers extracted the data, and the risk of bias was assessed using the Cochrane Manual 5.3. The meta-analysis was performed using RevMan 5.3. This review followed PRISMA 2020 and was registered in INPLASY (registration ID INPLASY202590085).

**Results:**

Following PRISMA guidelines, 2,136 records were identified, and five controlled studies involving 523 participants were included. Meta-analysis of progression-free survival (PFS) suggested a potential improvement in the targeted therapy plus chemotherapy group; however, the pooled result did not reach statistical significance (HR = 0.45, 95% CI 0.16–1.26; P = 0.13). Overall survival (OS) data were insufficient for quantitative meta-analysis, and the available controlled studies did not demonstrate a definitive survival benefit. The objective response rate (ORR) and disease control rate (DCR) were significantly higher with targeted therapy plus chemotherapy than with chemotherapy alone (P < 0.05). No significant increase in adverse events was observed.

**Conclusion:**

Targeted therapy plus chemotherapy improves tumor response outcomes in patients with advanced or metastatic TNBC without a clear increase in severe adverse events. However, evidence regarding PFS and OS benefits remains inconclusive because of limited studies, heterogeneity, and insufficient survival data.

## 1 Introduction

Triple-negative breast cancer (TNBC) is a biologically aggressive breast cancer subtype characterized by the absence of estrogen receptor, progesterone receptor, and human epidermal growth factor receptor 2 expression. It accounts for approximately 15-25% of breast cancers and is associated with younger age at diagnosis, higher rates of visceral relapse, early distant metastasis, and inferior prognosis compared with hormone receptor-positive or HER2-positive disease (1, 2). Because endocrine therapy and HER2-targeted therapy are not applicable to TNBC, systemic chemotherapy has historically remained the core treatment strategy, particularly in the recurrent or metastatic setting.

The therapeutic landscape of advanced TNBC has changed with the introduction of immune checkpoint inhibitors, poly(ADP-ribose) polymerase (PARP) inhibitors, antibody-drug conjugates, and anti-angiogenic agents. These approaches are biologically plausible because TNBC is heterogeneous at the molecular level and may exhibit immune-enriched phenotypes, DNA-damage-repair defects, or angiogenesis-related signaling activation (3–6). Nevertheless, the benefit of any targeted agent is dependent on disease setting, line of therapy, biomarker status, drug class, and chemotherapy backbone. Therefore, evidence generated in early-stage adjuvant or neoadjuvant treatment cannot be assumed to apply directly to patients with advanced or metastatic disease.

In advanced TNBC, immune checkpoint inhibition combined with chemotherapy has improved outcomes in selected PD-L1-positive populations, while PARP inhibitors provide benefit in patients with germline BRCA1/2 mutations and antibody-drug conjugates have become important later-line therapies (7–11). Anti-angiogenic treatment is another rational strategy because vascular endothelial growth factor receptor signaling contributes to tumor angiogenesis and metastatic progression. Apatinib, an oral VEGFR-2 inhibitor, has been evaluated in several Chinese studies of advanced or metastatic TNBC, commonly in combination with capecitabine after prior therapy failure (12, 13).

Published systematic reviews have often focused on a single drug class or a single therapeutic setting. Such reviews provide important evidence but do not fully address the comparative question of whether adding a targeted agent to chemotherapy improves response or survival in advanced or metastatic TNBC across the limited controlled literature. The present systematic review therefore synthesized controlled clinical evidence comparing targeted therapy plus chemotherapy with chemotherapy alone in patients with advanced, recurrent, unresectable, or metastatic TNBC. The analysis emphasized clinical consistency, appropriate handling of time-to-event outcomes, and cautious interpretation of drug-class heterogeneity.

## 2 Methods

### 2.1 Literature sources and search strategy

A comprehensive computerized search was performed in PubMed, EMBASE, the Cochrane Library, ScienceDirect, China Biomedical Literature Database (CBM), Wanfang Database, China National Knowledge Infrastructure (CNKI), and VIP Database. The search covered January 1, 2010, to August 31, 2025. The final database search was conducted on August 31, 2025. No language restrictions were applied during the electronic database search. Grey literature, conference abstracts without full publications, clinical trial registries, and unpublished datasets were not searched because only studies with sufficient comparative clinical data and extractable outcomes were eligible for quantitative synthesis. Manual backward citation tracking of included studies and relevant reviews was performed to identify additional eligible publications. Search terms combined controlled vocabulary and free-text terms related to triple-negative breast cancer, targeted therapy, chemotherapy, apatinib, anti-angiogenic therapy, immune checkpoint inhibitors, MEK inhibitors, PARP inhibitors, objective response, survival, and adverse events. The detailed search strategy is provided in **Supplementary Table 1**.

This systematic review was conducted according to the PRISMA 2020 statement and its explanation and elaboration document (14, 15). The review was prospectively registered with the International Platform of Registered Systematic Review and Meta-analysis Protocols (INPLASY; registration ID INPLASY202590085; doi: 10.37766/inplasy2025.9.0085). The PRISMA 2020 checklist is provided as **Supplementary Table 2**.

### 2.2 Literature inclusion and exclusion criteria

Eligible studies met the following criteria: (1) the study enrolled patients with pathologically confirmed advanced, recurrent, unresectable, or metastatic TNBC; (2) the intervention group received targeted therapy combined with chemotherapy; (3) the comparator group received chemotherapy alone or placebo plus chemotherapy; (4) the study reported at least one prespecified outcome, including PFS, OS, ORR, DCR, or adverse events; and (5) the study had a comparative clinical design, including randomized trials, prospective controlled studies, or retrospective comparative cohorts. Studies conducted exclusively in the adjuvant or neoadjuvant setting for early-stage TNBC were excluded unless advanced or metastatic data were available separately. Reviews, meta-analyses, case reports, non-comparative series, duplicate publications, studies with unclear efficacy evaluation, and studies without extractable comparative data were excluded.

### 2.3 Quality assessment and data extraction

Two reviewers independently screened titles, abstracts, and full texts, extracted data, and assessed methodological quality. Disagreements were resolved through discussion with a third reviewer. Extracted variables included first author, year, country or region, study design, disease setting, treatment line, sample size, targeted drug class, chemotherapy backbone, response outcomes, survival outcomes, adverse events, and follow-up. Randomized studies were assessed using the Cochrane RoB 2 framework, and non-randomized studies were assessed using ROBINS-I. The certainty of evidence was considered qualitatively based on study limitations, inconsistency, indirectness, imprecision, and risk of bias rather than through a formal GRADE summary of findings assessment (16–19).

### 2.4 Statistical analysis

Time-to-event outcomes were summarized preferentially using HRs and 95% CIs. When HRs were not reported, median survival values, ranges, confidence intervals, P values, and Kaplan-Meier information were extracted for descriptive synthesis rather than being pooled as weighted mean differences. This approach follows accepted methods for meta-analysis of survival endpoints and avoids treating censored time-to-event outcomes as simple continuous variables (20, 21). For dichotomous outcomes, including ORR, DCR, and adverse events, pooled ORs with 95% CIs were calculated when comparable definitions and sufficient event data were available. Safety outcomes were analyzed separately according to toxicity grade and specific adverse-event categories when reported by at least two studies. Toxicities with substantial differences in reporting criteria, grading systems, or drug-specific mechanisms were summarized descriptively. Fixed-effect models were used when heterogeneity was low, and random-effects models were used when clinical or statistical heterogeneity was substantial (22). Heterogeneity was quantified using the Chi-squared test and I² statistic. In addition to statistical heterogeneity, clinical heterogeneity was assessed qualitatively according to targeted drug class, chemotherapy backbone, treatment line, biomarker information, and disease burden. Because only five studies were available, formal meta-regression was not feasible.

## 3 Results

### 3.1 Characteristics of included studies

**Figure 1:**
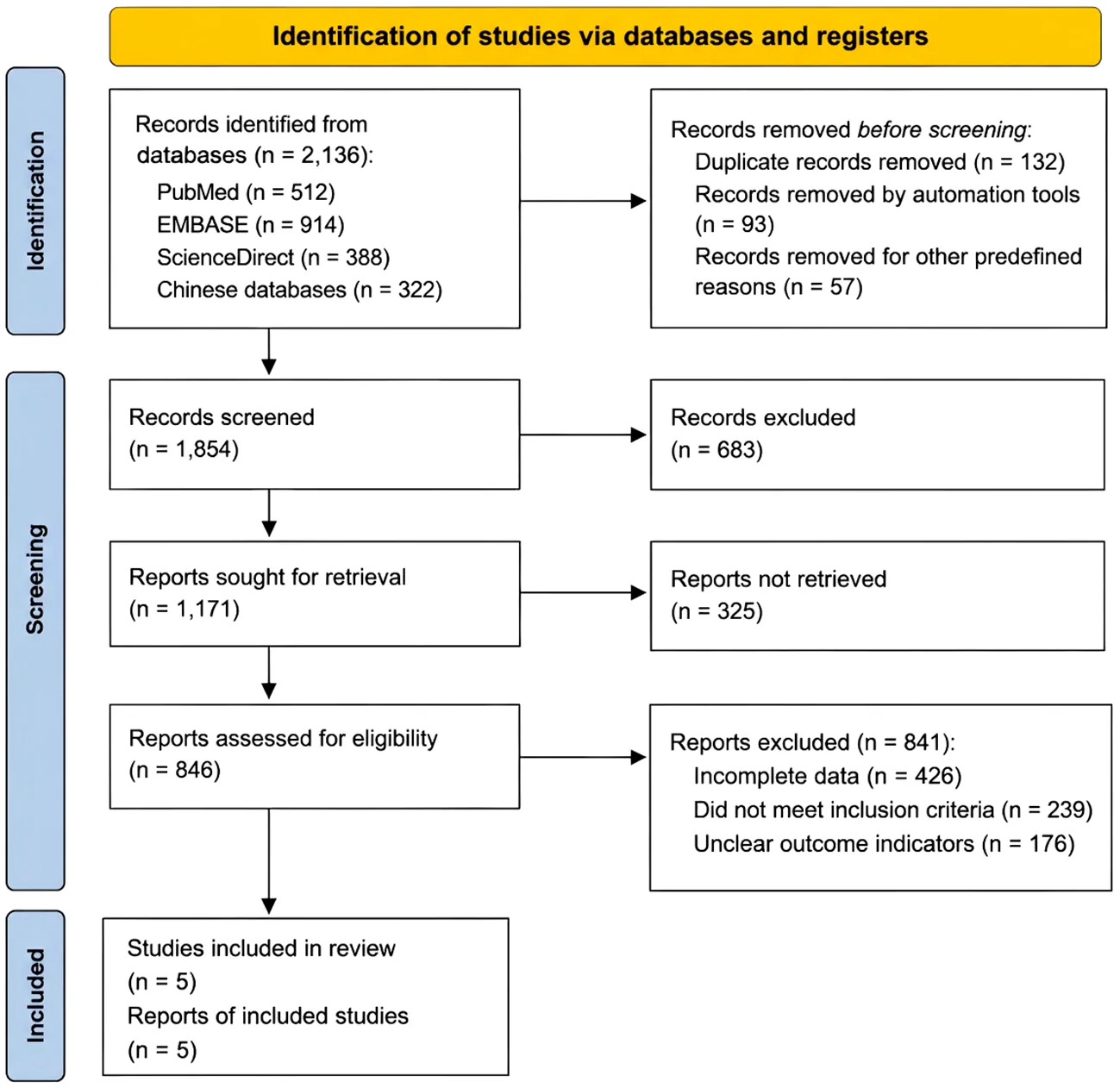
PRISMA 2020 flow diagram showing identification, screening, eligibility assessment, and inclusion of studies in the meta-analysis.

A total of 2,136 records were identified through database searches. Duplicate and automation-assisted eligibility screening left 1,854 records for title and abstract assessment. Of these, 683 were excluded at title and abstract screening, and 1,171 reports were sought for retrieval. After full-text review, 846 reports were assessed for eligibility. Five controlled studies involving 523 participants met the inclusion criteria and were included. The PRISMA 2020 flow diagram is shown in **Figure 1**, and the main characteristics of the included studies are summarized in **Table 1**.

**Table 1:** Basic characteristics of the included controlled studies.

Study	Year	Design/setting	Patients	Control	Experimental	Outcomes	Risk of bias
Brufsky A et al. (23)	2021	International multicenter phase II RCT; locally advanced/metastatic TNBC; first-line controlled cohort	C=43; T = 47	Placebo + paclitaxel	Cobimetinib + paclitaxel	PFS, OS, ORR, DCR, safety	Some concerns
Song XD et al. (24)	2023	Single-center randomized study by drawing lots; advanced TNBC	C=61; T = 62; response-evaluable denominator=48 per group	Capecitabine	Low-dose apatinib + capecitabine	ORR, DCR, tumor markers, safety	Some concerns
Wang Y et al. (25)	2021	Single-center comparative study; advanced metastatic TNBC after multiline treatment failure	C=52; T = 52	Capecitabine	Apatinib + capecitabine	PFS, ORR, DCR, safety	Serious confounding risk
Wang AJ et al. (26)	2020	Prospective randomized controlled study; recurrent/metastatic TNBC after second-line failure	C=30; T = 30	Capecitabine	Apatinib + capecitabine	PFS, OS, ORR, DCR, safety	Some concerns
Meng HR et al. (27)	2019	Retrospective comparative cohort; advanced TNBC after first- and second-line failure	C=73; T = 73	Capecitabine	Apatinib + capecitabine	PFS, ORR, DCR, safety	Serious confounding risk

C, control group; T, targeted therapy plus chemotherapy group; PFS, progression-free survival; OS, overall survival; ORR, objective response rate; DCR, disease control rate; TNBC, triple-negative breast cancer.

The included studies were published between 2019 and 2023. One international randomized phase II trial contributed a controlled cohort comparing cobimetinib plus paclitaxel with placebo plus paclitaxel in first-line locally advanced or metastatic TNBC (23). The remaining four studies were conducted in China and evaluated apatinib plus capecitabine compared with capecitabine alone in advanced, metastatic, or third-line TNBC (24–27). No included study evaluated adjuvant chemotherapy for early-stage curable disease as the primary treatment setting. The extracted outcome data for survival, tumor response, and safety endpoints are summarized in **Table 2**.

**Table 2:** Extracted outcome data from the included controlled studies.

Study	PFS	OS	ORR	DCR	Safety summary
Brufsky A et al. (23)	Median 5.5 vs 3.8 months; HR 0.73 (95% CI 0.43-1.24); P = 0.25	Median 16.0 vs 19.6 months; HR 1.05 (95% CI 0.55-2.01)	18/47 vs 9/43	36/47 vs 25/43	Serious AEs 17/47 vs 9/43; no grade 5 AEs in controlled cohort
Song XD et al. (24)	Not reported as HR or median PFS	Not reported	14/48 vs 8/48	31/48 vs 18/48	Grade III-IV hypertension, nausea/vomiting, leukopenia, and proteinuria were similar between groups
Wang Y et al. (25)	Median 6.00 months (95% CI 4.922-7.085) vs 4.00 months (95% CI 3.109-5.244); P = 0.004	Not reported	28/52 vs 13/52	44/52 vs 27/52	Grade III-IV hypertension, neutropenia, fatigue, vomiting, and hand-foot syndrome showed no significant between-group difference
Wang AJ et al. (26)	Median 7.0 vs 5.0 months; P<0.001	Median 13.0 vs 12.0 months; P = 0.258	8/30 vs 2/30	26/30 vs 18/30	Most AEs were grade I-II; grade III-IV hypertension was not increased; toxicity was manageable
Meng HR et al. (27)	HR 0.258 (95% CI 0.115-0.580); P = 0.002	Not reported	31/73 vs 15/73	51/73 vs 28/73	Most AEs were grade I-II; grade III-IV adverse events were not significantly different between groups

Values are reported as targeted therapy plus chemotherapy versus chemotherapy alone. Response-evaluable denominators were used when the original study reported response data on a subset of enrolled patients. HRs were pooled only when reported or directly extractable.

### 3.2 Quality assessment of included studies

**Figure 2:**
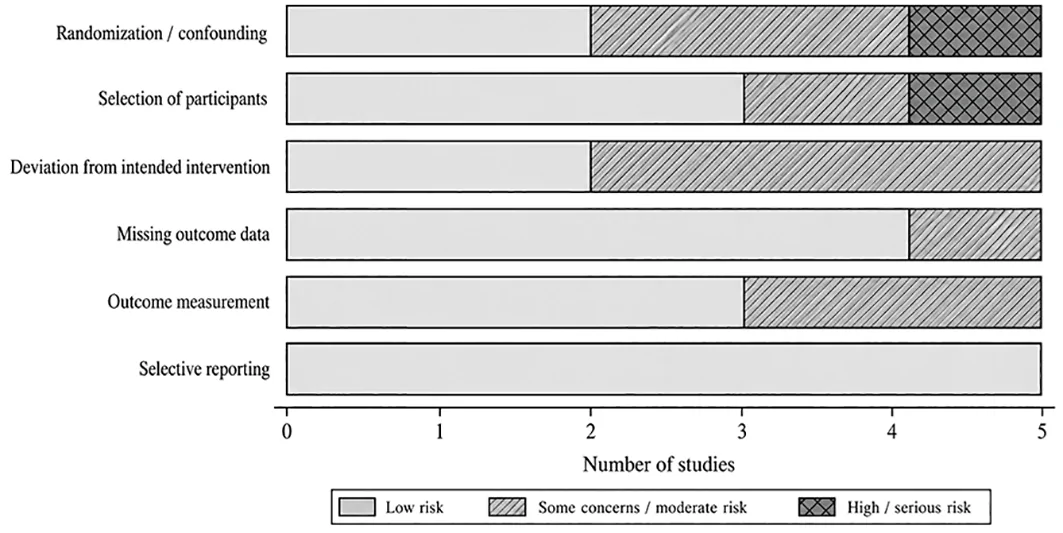
Risk-of-bias summary across included studies. Randomized studies were assessed using RoB 2, and non-randomized studies were assessed using ROBINS-I.

**Figure 3:**
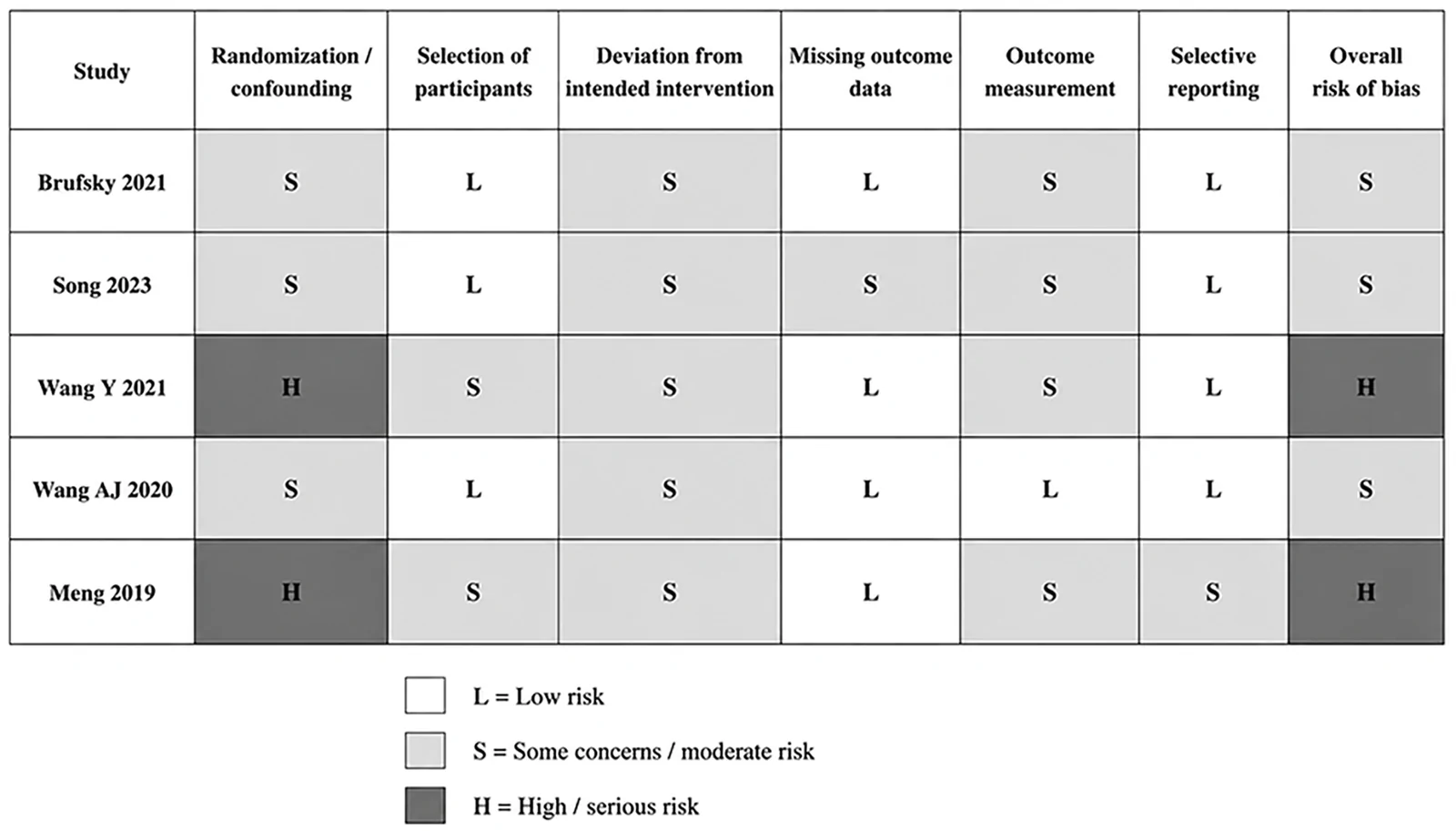
Study-level risk-of-bias matrix. L indicates low risk, S indicates some concerns or moderate risk, and H indicates high or serious risk.

Overall methodological quality was limited by small sample size and incomplete reporting of allocation concealment, blinding, and attrition. The international randomized phase II trial and the prospective randomized Chinese study had some concerns rather than low risk because of limited blinding details and small samples. Two retrospective or treatment-choice-based studies had serious or moderate risk of confounding. The risk-of-bias summary is shown in **Figure 2**, and study-level judgments are shown in **Figure 3**.

#### 3.2.1 Qualitative subgroup comparison according to targeted drug class and treatment setting

Given the limited number of eligible studies, formal subgroup meta-analysis was not performed because statistical comparisons between drug classes would have insufficient power and may generate unstable estimates. Instead, a structured qualitative comparison was conducted.

Among the four apatinib-based studies, all patients received capecitabine as the chemotherapy backbone and were generally treated in advanced or later-line settings after previous systemic therapy failure. These studies consistently reported improved ORR and DCR with apatinib plus capecitabine compared with capecitabine alone. Median PFS improvements ranged from approximately 2 to 3 months in studies reporting survival data.

In contrast, the COLET trial evaluated cobimetinib plus paclitaxel in a first-line locally advanced or metastatic TNBC population. Although response outcomes favored the combination strategy, the available controlled data did not demonstrate a significant improvement in PFS or OS.

These differences suggest that the magnitude of benefit from targeted therapy may depend on drug class, treatment line, and chemotherapy backbone.

### 3.3 Meta-analysis results

#### 3.3.1 Progression-free survival

**Figure 4:**
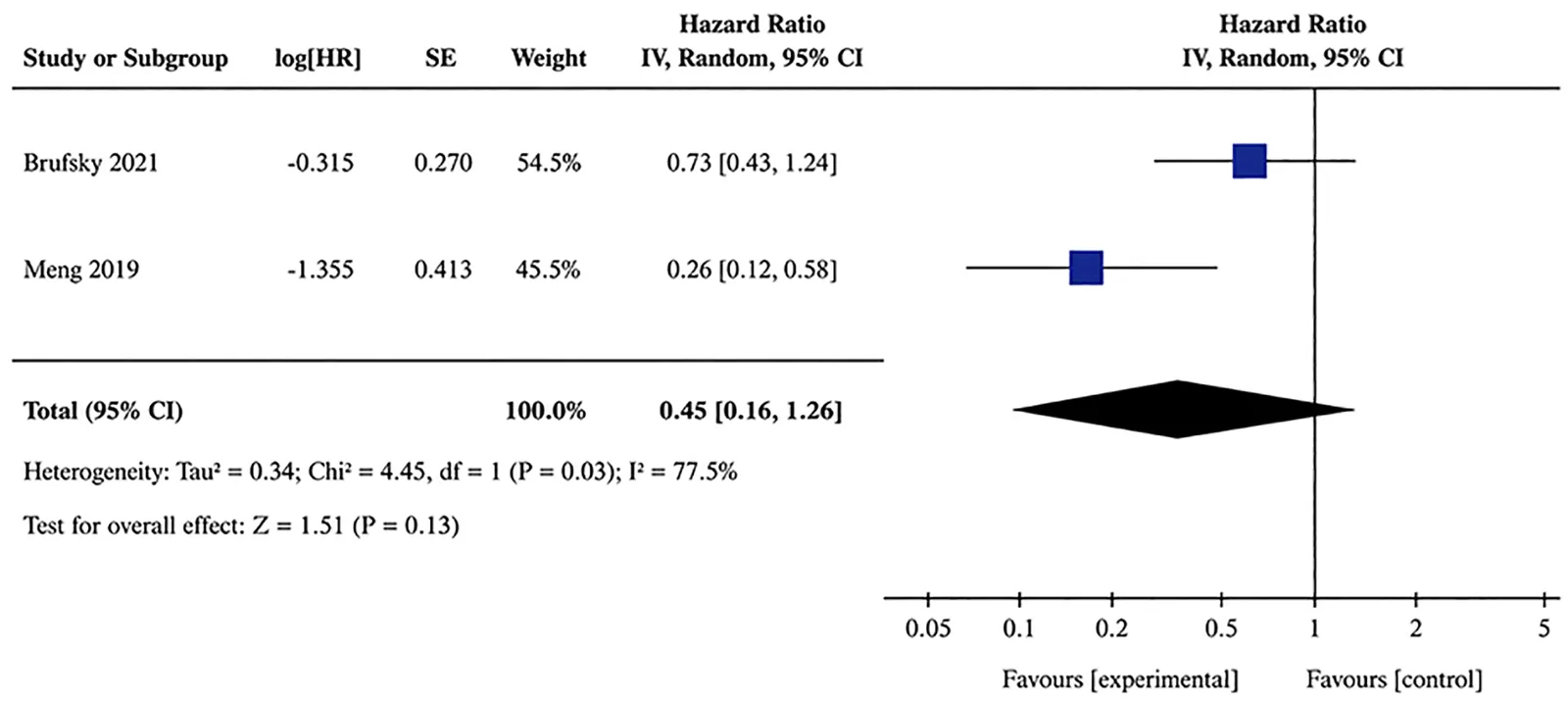
Exploratory forest plot of progression-free survival hazard ratios from studies with available HR estimates. Only two studies provided extractable HRs; therefore, quantitative pooling of four studies was not feasible.

PFS was reported inconsistently across studies. HRs were available from two studies: the COLET controlled cohort reported an HR of 0.73 (95% CI 0.43-1.24), and Meng et al. reported an HR of 0.258 (95% CI 0.115-0.580) (23, 27). A random-effects synthesis of these two HRs yielded a pooled HR of 0.45 (95% CI 0.16-1.26; P = 0.13), with substantial heterogeneity (I² = 77.5%). The PFS forest plot is shown in **Figure 4**. Other studies reported longer median PFS with apatinib plus capecitabine than with capecitabine alone, including 6.00 versus 4.00 months in Wang et al. and 7.0 versus 5.0 months in Wang AJ et al., but HRs were not available for direct pooling (25, 26). Therefore, PFS evidence was considered suggestive but statistically uncertain.

Although four studies reported PFS-related information, only two studies provided hazard ratios suitable for quantitative pooling. The remaining studies reported median PFS values without sufficient statistical information for HR reconstruction. Therefore, a four-study HR-based forest plot could not be generated without introducing unreliable assumptions. Median PFS comparisons from individual studies were summarized descriptively.

#### 3.3.2 Overall survival

OS data were insufficient for meta-analysis. The COLET controlled cohort reported median OS of 16.0 months with cobimetinib plus paclitaxel versus 19.6 months with placebo plus paclitaxel, with an HR of 1.05 (95% CI 0.55-2.01), indicating no OS advantage (23). Wang AJ et al. reported median OS of 13.0 months in the apatinib plus capecitabine group and 12.0 months in the capecitabine group, with no statistically significant difference (P = 0.258) (26). Because only one study reported an OS HR and treatment classes differed, OS was summarized descriptively. The available controlled data did not support a definitive OS benefit.

#### 3.3.3 Objective response rate

All five studies reported tumor response. The pooled OR for ORR was 2.83 (95% CI 1.87-4.28; P < 0.001; I² = 0%), favoring targeted therapy plus chemotherapy. Individual effects were directionally consistent across the cobimetinib and apatinib studies. The ORR forest plot is provided as **Supplementary Figure 1**.

#### 3.3.4 Disease control rate

DCR was extractable from all five studies after using response-evaluable denominators where appropriate. The pooled OR for DCR was 3.51 (95% CI 2.37-5.18; P < 0.001; I² = 0%), favoring targeted therapy plus chemotherapy. The DCR forest plot is shown in **Supplementary Figure 2**.

#### 3.3.5 Safety

Adverse-event reporting varied across studies because different grading systems were used, including CTCAE and WHO acute and subacute toxicity criteria. The available data did not show a consistent increase in overall severe adverse events with targeted therapy plus chemotherapy. However, toxicity profiles differed by targeted drug class. In the COLET controlled cohort, diarrhea, nausea, and rash were more common with cobimetinib plus paclitaxel than with placebo plus paclitaxel, and serious adverse events occurred in 17 of 47 patients versus 9 of 43 patients, with no grade 5 fatal adverse events in cohort I (23). In apatinib studies, hypertension, hand-foot syndrome, fatigue, and hematologic events were common, but grade III-IV events were generally manageable and did not consistently lead to treatment discontinuation (24–27). Exploratory pooled analyses of grade I–II and grade III–IV adverse events were performed when comparable event definitions were available. For grade III–IV toxicities, pooled analyses by individual toxicity category were explored when at least two studies reported comparable outcomes. No significant differences were observed for major reported severe toxicities, including hypertension (OR = 1.10, 95% CI 0.67–1.82), neutropenia (OR = 1.07, 95% CI 0.64–1.80), and hand-foot syndrome (OR = 1.14, 95% CI 0.42–3.13). The overall pooled analysis of grade III–IV adverse events showed no statistically significant increase with targeted therapy plus chemotherapy (OR = 1.02, 95% CI 0.77–1.36). However, these findings should be interpreted cautiously because adverse-event definitions, reporting completeness, and toxicity mechanisms differed between MEK inhibition and VEGFR inhibition. **Supplementary Figure 3** presents grade I–II adverse events. Forest plots of overall and toxicity-specific grade III–IV adverse events are presented in **Supplementary Figures 4**, **5**, respectively.

#### 3.3.6 Publication bias

Because each outcome included fewer than ten studies, formal funnel-plot asymmetry testing was not conducted. The small number of studies, the predominance of single-country apatinib studies, and the limited availability of unpublished negative studies were considered important qualitative sources of potential publication and selection bias.

#### 3.3.7 Capecitabine-based subgroup analysis

Because four of the five included studies used capecitabine as the chemotherapy backbone, an exploratory subgroup analysis was performed among these studies to evaluate the effect of apatinib-based targeted therapy in a more clinically homogeneous population.

In the capecitabine-based subgroup (4 studies, 473 patients), targeted therapy plus capecitabine significantly improved ORR compared with capecitabine alone (OR = 3.12, 95% CI 1.95–4.99, P < 0.001; I² = 0%). Similarly, DCR was significantly improved (OR = 3.86, 95% CI 2.34–6.36, P < 0.001; I² = 0%).

Survival outcomes remained difficult to synthesize because only two capecitabine-based studies reported extractable PFS information and OS data were incompletely reported.

## 4 Discussion

This systematic review and meta-analysis examined whether adding a targeted agent to chemotherapy confers additional benefit over chemotherapy alone in advanced or metastatic TNBC. All included studies enrolled patients with advanced, recurrent, unresectable, or metastatic disease; none involved adjuvant treatment for early-stage breast cancer. Therefore, the findings apply to the palliative setting and should not be extrapolated to curative-intent therapy.

The primary finding is that targeted therapy plus chemotherapy significantly improved objective response and disease control rates. This effect was directionally consistent across all five included studies and remained robust in the capecitabine-based subgroup analysis of four apatinib trials. The observation is biologically plausible: chemotherapy may enhance tumor antigen release or cytotoxic stress, while targeted agents may suppress resistance pathways or tumor angiogenesis, potentially creating synergistic antitumor activity.

However, this response benefit must be interpreted with substantial caution because the pooled analysis combined two mechanistically distinct classes of targeted agents. Four studies evaluated apatinib, a VEGFR-2 inhibitor, combined with capecitabine in heavily pretreated Chinese patients with advanced TNBC. One study evaluated cobimetinib, a MEK inhibitor, combined with paclitaxel in the first-line setting. These agents differ fundamentally in their molecular targets, biological rationale, patient populations, and therapeutic context. Thus, the pooled ORR and DCR estimates should be viewed as an exploratory synthesis of adding targeted therapy to chemotherapy rather than as evidence that different drug classes achieve equivalent efficacy.

Notably, improved tumor response did not translate into a clear survival advantage. Progression-free survival hazard ratios were extractable from only two studies, and the pooled estimate was not statistically significant with considerable heterogeneity. Overall survival data were insufficient for quantitative synthesis, and the available controlled comparisons did not demonstrate a definitive OS benefit. This discrepancy between response and survival outcomes is not uncommon in later-line cancer trials, where post-progression therapies and patient heterogeneity may attenuate survival signals despite early tumor shrinkage.

The heterogeneity across studies extends beyond drug class to include differences in treatment line, chemotherapy backbone, biomarker selection, and disease burden. These factors likely influence both the magnitude of treatment effect and its generalizability. The findings are broadly consistent with previous reviews showing that targeted treatment effects in TNBC are highly dependent on drug class, biomarker selection, and treatment setting (28–31). Current ESMO and NCCN guidelines emphasize biomarker-driven treatment selection in metastatic TNBC, recommending immune checkpoint inhibitors for PD-L1-positive disease, PARP inhibitors for patients with germline BRCA1/2 mutations, and antibody-drug conjugates for appropriately selected later-line patients (32, 33). These recommendations reflect the recognition that TNBC is not a single uniform entity but comprises diverse molecular subtypes—basal-like, immune-enriched, mesenchymal-related, and others—that may differentially respond to specific targeted approaches (5, 6).

Immune checkpoint inhibitors combined with chemotherapy have demonstrated survival benefit in selected PD-L1-positive populations, whereas PARP inhibitors provide clinically meaningful benefit in patients with homologous recombination repair defects (7–10). More recently, antibody-drug conjugates, including sacituzumab govitecan, have become important treatment options after chemotherapy exposure (11). By contrast, the present analysis included no studies of immune checkpoint inhibitors or PARP inhibitors as the targeted component; the evidence base was dominated by apatinib plus capecitabine studies. This limits the ability to draw conclusions about the broader class of targeted therapies and underscores the importance of distinguishing between drug classes rather than treating them as interchangeable.

The external validity of our findings warrants particular consideration. Four of the five included studies originated from China and enrolled predominantly Chinese patients receiving apatinib plus capecitabine after multiple prior therapies. While these data provide valuable evidence for this specific clinical context, their applicability to European, North American, and other multiethnic populations remains uncertain. Differences in genetic background, biomarker prevalence, treatment accessibility, subsequent therapy options, and supportive care practices may influence both efficacy and toxicity profiles. The limited representation of non-Asian populations precludes reliable assessment of global generalizability.

Regarding safety, the available data did not show a consistent increase in severe adverse events with combination therapy. However, this should not be interpreted as evidence of uniform tolerability. Toxicity profiles were drug-class specific: MEK inhibition was associated with gastrointestinal and dermatologic adverse effects in the COLET trial, whereas apatinib-based regimens more commonly caused hypertension, hand-foot syndrome, and fatigue. These class-specific patterns require proactive monitoring and management rather than assuming the combination is uniformly safe across all targeted agents. Future studies should report standardized grade-specific adverse events and treatment discontinuation rates to permit more reliable safety meta-analysis.

Several limitations constrain the interpretation of these results. First, only five controlled studies met eligibility criteria over a 15-year search period, yielding a modest total sample size. Second, the included studies varied substantially in disease setting, treatment line, drug class, chemotherapy backbone, and outcome reporting. Third, survival endpoints were incompletely reported as hazard ratios, limiting valid time-to-event meta-analysis. Fourth, the predominance of single-country, small-sample Chinese studies restricts geographic diversity and generalizability. Fifth, formal publication bias assessment was not feasible because the number of studies per outcome was too small. Sixth, the overall certainty of evidence is limited by small sample sizes, clinical heterogeneity, incomplete reporting of survival data, and risk of bias. A formal GRADE Summary of Findings assessment was not performed due to the limited number of included studies and outcomes.

Despite these limitations, this review provides a clinically coherent synthesis of the available controlled evidence. The analysis supports the hypothesis that targeted therapy plus chemotherapy can improve tumor response in advanced or metastatic TNBC, particularly in the context of apatinib-based capecitabine treatment in later-line settings. However, consistent with current ESMO and NCCN recommendations that emphasize biomarker-guided therapy selection (32, 33), these findings should not be interpreted as support for routine use of all targeted agents across unselected TNBC populations. The results underscore the need for adequately powered randomized trials stratified by targeted mechanism, biomarker status, and treatment line, with standardized reporting of hazard ratios for survival outcomes.

## 5 Conclusion

Targeted therapy plus chemotherapy was associated with improved objective response and disease control rates in patients with advanced or metastatic TNBC, especially in capecitabine-based apatinib studies. However, because the available evidence includes substantial heterogeneity in drug mechanism, treatment line, chemotherapy backbone, and biomarker context, the findings should be interpreted as hypothesis-generating rather than definitive evidence of a universal targeted therapy benefit. Evidence for progression-free and overall survival benefits remains inconclusive due to the small number of studies, substantial heterogeneity, incomplete hazard ratio reporting, and differences in drug class and treatment line. The safety data do not indicate a consistent increase in severe adverse events, but class-specific toxicities require careful monitoring. These findings support further randomized, biomarker-stratified trials rather than routine extrapolation across all targeted therapies or disease settings.

## Data Availability

The original contributions presented in the study are included in the article/**Supplementary Material**. Further inquiries can be directed to the corresponding author.

## Supplementary material

Supplementary Figure 1 RevMan-style forest plot of objective response rate comparing targeted therapy plus chemotherapy with chemotherapy alone.

Supplementary Figure 2 RevMan-style forest plot of disease control rate comparing targeted therapy plus chemotherapy with chemotherapy alone.

Supplementary Figure 3 Forest plot of grade I–II adverse events comparing targeted therapy plus chemotherapy versus chemotherapy alone. Mild toxicities were analyzed separately because targeted agents may produce class-specific low-grade adverse events despite no increase in severe toxicity.

Supplementary Figure 4 Forest plot of grade III–IV adverse events comparing targeted therapy plus chemotherapy versus chemotherapy alone. Comparable adverse-event categories reported in at least two studies were included. The pooled analysis showed no significant increase in severe toxicities with targeted therapy addition (OR = 1.02, 95% CI 0.77–1.36). However, toxicity patterns differed according to targeted drug class.

Supplementary Figure 5 Forest plots of grade III–IV adverse events according to individual toxicity categories. **(A)** Hypertension III–IV. **(B)** Neutropenia III–IV. **(C)** Hand-foot syndrome III–IV.
