# Supplementary material for: Targeted therapy plus chemotherapy for advanced or metastatic triple-negative breast cancer: a PRISMA 2020 systematic review and meta-analysis
Source: Front Oncol. 2026 Aug 14;16:1903922. doi: 10.3389/fonc.2026.1903922 (PMC13521797; doi:10.3389/fonc.2026.1903922)
Supplement: Supplementary file 1 [file Table1.docx]

**Supplementary Table S1 Detailed search strategy**

| **Database/source** | **Search strategy** | **Limits/filters** |
| --- | --- | --- |
| PubMed | ("triple-negative breast cancer"[Title/Abstract] OR "triple negative breast cancer"[Title/Abstract] OR TNBC[Title/Abstract] OR "Triple Negative Breast Neoplasms"[MeSH Terms]) AND ("targeted therapy"[Title/Abstract] OR "molecular targeted therapy"[Title/Abstract] OR apatinib[Title/Abstract] OR cobimetinib[Title/Abstract] OR "MEK inhibitor"[Title/Abstract] OR "anti-angiogenic"[Title/Abstract] OR "angiogenesis inhibitor"[Title/Abstract] OR "immune checkpoint inhibitor"[Title/Abstract] OR atezolizumab[Title/Abstract] OR pembrolizumab[Title/Abstract] OR "PARP inhibitor"[Title/Abstract] OR olaparib[Title/Abstract] OR talazoparib[Title/Abstract]) AND (chemotherapy[Title/Abstract] OR capecitabine[Title/Abstract] OR paclitaxel[Title/Abstract] OR taxane[Title/Abstract]) AND (advanced[Title/Abstract] OR metastatic[Title/Abstract] OR recurrent[Title/Abstract] OR unresectable[Title/Abstract]) AND (randomized[Title/Abstract] OR trial[Title/Abstract] OR controlled[Title/Abstract] OR cohort[Title/Abstract] OR comparative[Title/Abstract]) | Publication date: January 1, 2010 to August 31, 2025; humans; English or Chinese articles when available. |
| EMBASE | ('triple negative breast cancer'/exp OR 'triple negative breast cancer':ti,ab OR tnbc:ti,ab) AND ('targeted therapy'/exp OR 'molecular targeted therapy':ti,ab OR apatinib:ti,ab OR cobimetinib:ti,ab OR 'mek inhibitor':ti,ab OR 'antiangiogenic therapy':ti,ab OR 'immune checkpoint inhibitor':ti,ab OR atezolizumab:ti,ab OR pembrolizumab:ti,ab OR 'parp inhibitor':ti,ab OR olaparib:ti,ab OR talazoparib:ti,ab) AND ('chemotherapy'/exp OR chemotherapy:ti,ab OR capecitabine:ti,ab OR paclitaxel:ti,ab OR taxane:ti,ab) AND (advanced:ti,ab OR metastatic:ti,ab OR recurrent:ti,ab OR unresectable:ti,ab) AND (randomized:ti,ab OR trial:ti,ab OR controlled:ti,ab OR cohort:ti,ab OR comparative:ti,ab) | Publication years 2010-2025; human studies; English or Chinese articles when available. |
| Cochrane Library | (("triple-negative breast cancer" OR "triple negative breast cancer" OR TNBC) AND (targeted OR apatinib OR cobimetinib OR "MEK inhibitor" OR anti-angiogenic OR "immune checkpoint inhibitor" OR atezolizumab OR pembrolizumab OR "PARP inhibitor" OR olaparib OR talazoparib) AND (chemotherapy OR capecitabine OR paclitaxel OR taxane) AND (advanced OR metastatic OR recurrent OR unresectable)) | Trials and reviews searched; publication date 2010-2025. |
| ScienceDirect | "triple-negative breast cancer" OR TNBC AND targeted therapy AND chemotherapy AND (advanced OR metastatic OR recurrent) AND (apatinib OR cobimetinib OR anti-angiogenic OR immune checkpoint inhibitor OR PARP inhibitor) | Article title, abstract, and keywords; publication date 2010-2025. |
| China Biomedical Literature Database (CBM) | (三阴性乳腺癌 OR 三阴乳腺癌 OR TNBC) AND (靶向治疗 OR 分子靶向 OR 阿帕替尼 OR 科比替尼 OR MEK抑制剂 OR 抗血管生成 OR 免疫检查点抑制剂 OR PARP抑制剂) AND (化疗 OR 卡培他滨 OR 紫杉醇) AND (晚期 OR 转移 OR 复发 OR 不可切除) | Publication date: 2010-2025; clinical studies; Chinese or English. |
| Wanfang Database | (三阴性乳腺癌 OR 三阴乳腺癌 OR TNBC) AND (靶向治疗 OR 阿帕替尼 OR 科比替尼 OR 抗血管生成 OR 免疫治疗 OR PARP抑制剂) AND (化疗 OR 卡培他滨 OR 紫杉醇) AND (晚期 OR 转移 OR 复发 OR 不可切除) | Publication date: 2010-2025; journal articles and dissertations screened when relevant. |
| China National Knowledge Infrastructure (CNKI) | (三阴性乳腺癌 OR 三阴乳腺癌 OR TNBC) AND (靶向治疗 OR 分子靶向 OR 阿帕替尼 OR 抗血管生成 OR 免疫检查点抑制剂 OR PARP抑制剂) AND (化疗 OR 卡培他滨 OR 紫杉醇) AND (晚期 OR 转移 OR 复发 OR 不可切除) | Publication date: 2010-2025; clinical comparative studies screened. |
| VIP Database | (三阴性乳腺癌 OR 三阴乳腺癌 OR TNBC) AND (靶向治疗 OR 阿帕替尼 OR 抗血管生成 OR 免疫治疗 OR PARP抑制剂) AND (化疗 OR 卡培他滨 OR 紫杉醇) AND (晚期 OR 转移 OR 复发 OR 不可切除) | Publication date: 2010-2025; journal articles screened. |
| Manual search | Backward citation tracking of included studies and relevant reviews; duplicate reports and non-comparative records were removed during screening. | Studies were eligible only if they provided extractable comparative data for targeted therapy plus chemotherapy versus chemotherapy alone or placebo plus chemotherapy in advanced, recurrent, unresectable, or metastatic TNBC. |
