# Supplementary material for: Targeted therapy plus chemotherapy for advanced or metastatic triple-negative breast cancer: a PRISMA 2020 systematic review and meta-analysis
Source: Front Oncol. 2026 Aug 14;16:1903922. doi: 10.3389/fonc.2026.1903922 (PMC13521797; doi:10.3389/fonc.2026.1903922)
Supplement: Supplementary file 2 [file Table2.docx]

**Supplementary Table S2 PRISMA 2020 checklist**

| **Section/topic** | **Item** | **PRISMA 2020 checklist item** | **Reported in the manuscript** |
| --- | --- | --- | --- |
| Title | 1 | Identify the report as a systematic review. | Title |
| Abstract | 2 | Provide a structured summary including background, objectives, eligibility criteria, information sources, risk of bias, synthesis methods, results, limitations, interpretation, and registration. | Abstract |
| Introduction | 3 | Describe the rationale for the review in the context of existing knowledge. | Introduction |
| Introduction | 4 | Provide an explicit statement of the objective or question addressed by the review. | Abstract; Introduction |
| Methods | 5 | Specify the inclusion and exclusion criteria for the review and how studies were grouped for the syntheses. | Methods 2.2 |
| Methods | 6 | Specify all databases, registers, websites, organizations, reference lists, and other sources searched or consulted, and specify the date when each source was last searched or consulted. | Methods 2.1; Supplementary Table S1 |
| Methods | 7 | Present the full search strategies for all databases, registers, and websites, including any filters and limits used. | Supplementary Table S1 |
| Methods | 8 | Specify the methods used to decide whether each study met the inclusion criteria, including how many reviewers screened records and reports. | Methods 2.3; Results 3.1 |
| Methods | 9 | Specify the methods used to collect data from reports, including how many reviewers collected data and how disagreements were resolved. | Methods 2.3 |
| Methods | 10a | List and define all outcomes for which data were sought, and specify whether all results compatible with each outcome domain were sought. | Methods 2.2-2.4 |
| Methods | 10b | List and define all other variables for which data were sought. | Methods 2.3 |
| Methods | 11 | Specify the methods used to assess risk of bias in the included studies, including how many reviewers assessed each study and how disagreements were resolved. | Methods 2.3 |
| Methods | 12 | Specify the effect measures used for each outcome. | Methods 2.4 |
| Methods | 13a | Describe the processes used to decide which studies were eligible for each synthesis. | Methods 2.2-2.4; Results 3.3 |
| Methods | 13b | Describe any methods required to prepare the data for presentation or synthesis, such as handling missing summary statistics or data conversions. | Methods 2.4; Table 2 note |
| Methods | 13c | Describe any methods used to tabulate or visually display results of individual studies and syntheses. | Methods 2.4; Figures 1-4; Supplementary Figures S1-S9 |
| Methods | 13d | Describe any methods used to synthesize results and provide a rationale for model choice. | Methods 2.4 |
| Methods | 13e | Describe any methods used to explore possible causes of heterogeneity among study results. | Methods 2.4; Discussion |
| Methods | 13f | Describe any sensitivity analyses conducted to assess robustness of synthesized results. | Not conducted because of the small number of studies and limited HR reporting; discussed as a limitation |
| Methods | 14 | Describe any methods used to assess risk of bias due to missing results in a synthesis. | Methods 2.4; Results 3.3.6 |
| Methods | 15 | Describe any methods used to assess certainty or confidence in the body of evidence for each outcome. | Methods 2.3; Discussion |
| Results | 16a | Describe the results of the search and selection process, including numbers of records identified, screened, assessed for eligibility, and included. | Results 3.1; Figure 1 |
| Results | 16b | Cite studies that appeared to meet inclusion criteria but were excluded, and explain why they were excluded. | Results 3.1; Figure 1 |
| Results | 17 | Cite each included study and present its characteristics. | Results 3.1; Table 1 |
| Results | 18 | Present assessments of risk of bias for each included study. | Results 3.2; Figures 2-3; Table 1 |
| Results | 19 | For all outcomes, present summary statistics for each group and effect estimates for each included study. | Results 3.3; Table 2; Figure 4; Supplementary Figures S1-S4 |
| Results | 20a | For each synthesis, briefly summarize the characteristics and risk of bias among contributing studies. | Results 3.1-3.3; Discussion |
| Results | 20b | Present results of all statistical syntheses conducted, including summary estimates, confidence intervals, and heterogeneity statistics. | Results 3.3; Figure 4; Supplementary Figures S1-S4 |
| Results | 20c | Present results of investigations of possible causes of heterogeneity among study results. | Discussion; formal subgroup analysis was not conducted because of the small evidence base |
| Results | 20d | Present results of all sensitivity analyses conducted to assess robustness of synthesized results. | Not conducted; limitation reported in Discussion |
| Results | 21 | Present assessments of risk of bias due to missing results for each synthesis assessed. | Results 3.3.6; Supplementary Figures S5-S9 |
| Results | 22 | Present assessments of certainty or confidence in the body of evidence for each outcome assessed. | Discussion; certainty was limited by small sample size, heterogeneity, and incomplete HR reporting |
| Discussion | 23a | Provide a general interpretation of the results in the context of other evidence. | Discussion |
| Discussion | 23b | Discuss limitations of the evidence included in the review. | Discussion |
| Discussion | 23c | Discuss limitations of the review processes used. | Discussion |
| Discussion | 23d | Discuss implications of the results for practice, policy, and future research. | Discussion; Conclusion |
| Other information | 24a | Provide registration information for the review, including register name and registration number, or state that the review was not registered. | Methods 2.1; Clinical register number |
| Other information | 24b | Indicate where the review protocol can be accessed, or state that a protocol was not prepared. | Methods 2.1; registration record cited |
| Other information | 24c | Describe and explain any amendments to information provided at registration or in the protocol. | No amendments reported |
| Other information | 25 | Describe sources of financial or non-financial support and the role of funders or sponsors. | Funding Sources |
| Other information | 26 | Declare any competing interests of review authors. | Conflict of Interest Statement |
| Other information | 27 | Report which data, analytic code, and other materials are publicly available and where they can be found. | Data Availability Statement |
